# Sensitive Immunofluorescent Detection of the PRAME Antigen Using a Practical Antibody Conjugation Approach

**DOI:** 10.3390/ijms222312845

**Published:** 2021-11-27

**Authors:** Ksenia A. Sapozhnikova, Vsevolod A. Misyurin, Dmitry Y. Ryazantsev, Egor A. Kokin, Yulia P. Finashutina, Anastasiya V. Alexeeva, Igor A. Ivanov, Milita V. Kocharovskaya, Nataliya A. Tikhonova, Galina P. Popova, Vera A. Alferova, Alexey V. Ustinov, Vladimir A. Korshun, Vladimir A. Brylev

**Affiliations:** 1 Shemyakin-Ovchinnikov Institute of Bioorganic Chemistry, Miklukho-Maklaya 16/10, 117997 Moscow, Russia; ksapozh@mail.ru (K.A.S.); d.yu.ryasantsev@gmail.com (D.Y.R.); tujhxz09@mail.ru (E.A.K.); chai.mail0@gmail.com (I.A.I.); kocharovskaya.mv@phystech.edu (M.V.K.); gpopova305@gmail.com (G.P.P.); alferovava@gmail.com (V.A.A.); austinov@yandex.ru (A.V.U.); 2 N.N. Blokhin National Medical Research Center of Oncology, Kashirskoye Highway 23, 115478 Moscow, Russia; vsevolod.misyurin@gmail.com (V.A.M.); j_finashutina@mail.ru (Y.P.F.); 3 Faculty of General Medicine, Pirogov Russian National Research Medical University, Ostrovityanova 1, 117992 Moscow, Russia; nastval99@mail.ru; 4 Moscow Institute of Physics and Technology, Institutsky Lane 9, 141700 Dolgoprudny, Russia; 5 GeneTechnology LLC, Profsoyuznaya 104, 117437 Moscow, Russia; n.borodulina94@mail.ru; 6 Gause Institute of New Antibiotics, B. Pirogovskaya 11, 119021 Moscow, Russia

**Keywords:** antibodies, PRAME, oxime ligation, ethoxyethylidene protecting group, fluorescent dyes, fluorescence imaging

## Abstract

Bioconjugation of antibodies with various payloads has diverse applications across various fields, including drug delivery and targeted imaging techniques. Fluorescent immunoconjugates provide a promising tool for cancer diagnostics due to their high brightness, specificity, stability and target affinity. Fluorescent antibodies are widely used in flow cytometry for fast and sensitive identification and collection of cells expressing the target surface antigen. Nonetheless, current approaches to fluorescent labeling of antibodies most often use random modification, along with a few rather sophisticated site-specific techniques. The aim of our work was to develop a procedure for fluorescent labeling of immunoglobulin G via periodate oxidation of antibody glycans, followed by oxime ligation with fluorescent oxyamines. Here, we report a novel technique based on an in situ oxime ligation of ethoxyethylidene-protected aminooxy compounds with oxidized antibody glycans. The approach is suitable for easy modification of any immunoglobulin G, while ensuring that antigen-binding domains remain intact, thus revealing various possibilities for fluorescent probe design. The technique was used to label an antibody to PRAME, a cancer-testis protein overexpressed in a number of cancers. A 6H8 monoclonal antibody to the PRAME protein was directly modified with protected-oxyamine derivatives of fluorescein-type dyes (FAM, Alexa488, BDP-FL); the stoichiometry of the resulting conjugates was characterized spectroscopically. The immunofluorescent conjugates obtained were applied to the analysis of bone marrow samples from patients with oncohematological diseases and demonstrated high efficiency in flow cytometry quantification. The approach can be applied for the development of various immunofluorescent probes for detection of diagnostic and prognostic markers, which can be useful in anticancer therapy.

## 1. Introduction

There are a number of approaches to chemical modification of antibodies, especially with fluorescent dyes [1,2,3,4,5,6] and therapeutic agents [7,8,9,10,11,12]. Each method of bioconjugation has its advantages and disadvantages, which define its scope of application. For example, modification of lysine side chain NH_2_ groups does not provide regiospecificity, thus, excessive labeling of an antibody diminishes its affinity [2,3,6]. Partial reduction of disulfide bridges, followed by Michael-type modification of any arising thiol groups, may cause disulfide bond scrambling and break the native quaternary antibody structure [13,14,15]. In addition, there are many complex chemical and chemo-enzymatic techniques for site-specific antibody modification using intricate modification reagents [12,16,17,18,19,20].

Oxime ligation, a “click” reaction of oxyamines (*O*-substituted hydroxylamines) with aldehydes and ketones in an aqueous solution at physiological pH, is widely used as a tool for bioconjugation [21]. The oxime linkage is stable enough [22] to ensure the integrity of such bioconjugates in various applications. Carbonyl groups suitable for oxime ligation can be introduced into the antibody molecule by periodate oxidation of the glycosylated site [5,23,24], even one that has been genetically engineered [25]. Alternatively, the carbonyl group can be introduced into the antibody by enzymatic modification of the glycan part with a keto-sugar [26], or via site-specific incorporation of genetically encoded *p*-acetylphenylalanine into a peptide chain [27,28]. Whereas the two latter approaches require cumbersome enzymatic or genetic manipulations, periodate oxidation of the carbohydrate site can be easily applied to any full-size antibody. The glycosylation site of immunoglobulin G is located at the heavy chain, at a significant distance from the antigen binding domain, thus, the modification does not influence antibody affinity.

The obvious requirements for a fluorescent immunoconjugate (an antibody modified with fluorescent dyes) are high brightness, specificity, high chemical and spectral stability and the ability to preserve antibody affinity [5]. We aimed to develop a convenient procedure for labeling any immunoglobulin G via periodate oxidation followed by oxime ligation.

While some oxyamine derivatives of fluorescent dyes are commercially available, their high reactivity limits their storage time even in salt form, e.g., hydrochloride. Derivatives of luminescent dyes that are stable when stored with a masked oxyamine function and easily releasable before conjugation with carbonyl compounds are of potential interest. We aimed to develop fluorescent reagents with such properties, a procedure for their reaction with aldehydes, and to demonstrate the usefulness of the reagents for labeling of oxidized immunoglobulins. Here, we report the synthesis of fluorescent dye derivatives that contain an ethoxyethylidene-protected aminooxy function and their application to fluorescent labeling of antibodies.

To demonstrate the viability of the approach, we used an antibody to PRAME (preferentially expressed antigen in melanoma), a cancer-testis protein that is overexpressed in a number of cancers and is thought to be suitable as a diagnostic and prognostic marker in anticancer therapy [29,30,31,32,33,34,35,36,37,38,39,40,41]. Recently, we developed an approach for labeling an 6H8 monoclonal antibody to the PRAME protein with fluorescent dye Cy3 [42]. Here, we report the direct labeling of this antibody with protected-oxyamine derivatives of dyes with fluorescein-type emission.

## 2. Results and Discussion

### 2.1. Synthesis of Modifying Reagents

**Scheme 1:**
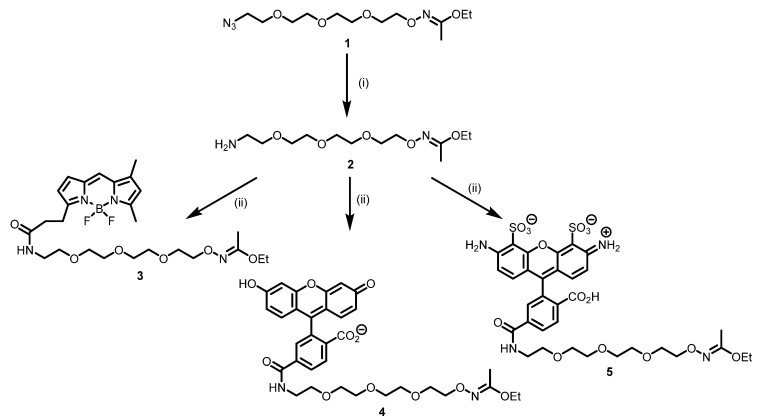
Synthesis of 1-ethoxyethylidene-protected oxyamine derivatives of fluorescent dyes. Reagents and conditions: (i) PPh_3_, THF, 2 h, then H_2_O; (ii) dye NHS-ester, DMF, DIPEA, 0 °C.

We found that the 1-Ethoxyethylidene group, used to protect O-alkylhydroxylamines, is completely stable under alkaline conditions and is easily removable under acidic conditions [43,44]. Our aim was to prepare derivatives of fluorescent dyes containing a protected oxyamine function. Bifunctional protected oxyamine-azide tetraethyleneglycol-based reagent **1** [42] was used as the starting compound. Selective azide reduction by Ph_3_P yielded bifunctional reagent **2,** containing amine and a masked oxyamine function. Compound **2** was acylated by oxysuccinimide esters of fluorescent dyes—BODIPY FL (BDP-FL), 6-carboxyfluorescein (FAM), and Alexa 488 (AF488)—to yield protected derivatives **3**–**5** (Scheme 1). Attempts to remove the protection group in compounds **3**–**5** were unsuccessful due to considerable degradation of the dyes in the presence of the free oxyamine function.

### 2.2. Modification of Monoclonal Antibody 6H8

The presence of the PRAME protein was observed on the surface of K562 leukemic cells, which suggested the possibility of using PRAME as a target for immunotherapy. Since PRAME expression is not limited to leukemia, monitoring its expression in solid tumor cells will enable the development of new drugs for these diseases as well. In our study, the presence of the PRAME protein on the surface of melanoma cells was assessed by flow cytometry. According to the results obtained, this protein is active on the surface of mel P cells, which are metastatic melanoma. The intensity of the staining of mel P and K562 cells was comparable. Therefore, the PRAME protein is located on the surface of melanoma cells and can be used as a target for immunotherapy.

**Figure 1:**
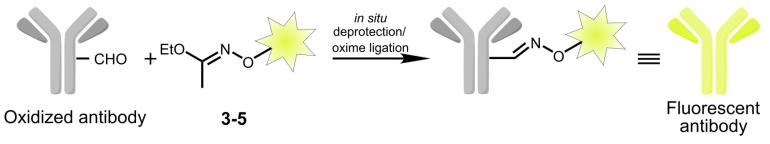
Antibody labeling scheme.

The modification of the 6H8 antibody was based on periodate oxidation, to generate aldehyde groups on the glycoside moieties of the immunoglobulin. The resulting oxidized antibodies were labeled by oxyamine dyes generated in situ from compounds **3**–**5** to yield fluorescent antibodies (Figure 1).

**Figure 2:**
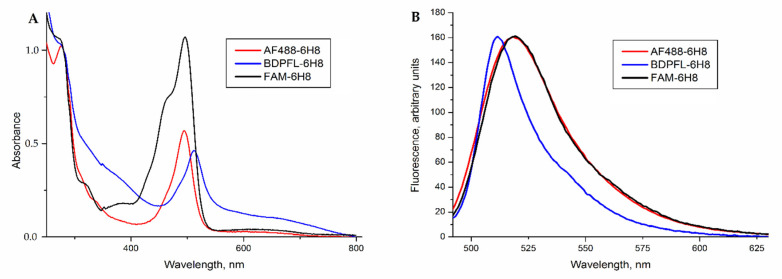
(**A**) Normalized absorption spectra of fluorescently labeled monoclonal antibody 6H8; (**B**) Normalized fluorescence spectra of fluorescently labeled monoclonal antibody 6H8 (λ_ex_ = 484 nm).

The absorption spectra of the labeled antibodies (Figure 2A) contain the characteristic absorption bands of all of the dyes used for modification. The correlation of absorption at 280 nm and at the characteristic wavelengths allows for the determination of the dye load. Fluorescent conjugates with dye-antibody ratios of 4 (FAM), 2 (BDP-FL) and 2.5 (AF488) were obtained.

The emission maxima (Figure 2B) of the fluorescent 6H8 conjugates had the same wavelengths as those of the initial dyes FAM and AF488 (520 nm), and BDP-FL (509 nm). Thus, the method developed provides a load of 2–4 fluorescent dye per antibody molecule. Fluorescein-type dyes retained their photophysical properties under these conditions.

### 2.3. PRAME Detection by Flow Cytometry

Our preliminary experiments PRAME protein detection, based on phycoerythrin-labeled secondary antibodies to the 6H8 monoclonal antibody, showed that the intensity of the fluorescent signal in flow cytometry was low in comparison with control samples. This method was complicated by two-stage binding, which led to an unstable labeling construct on the cell surface. As a result, we opted for a direct labeling strategy, using a covalently labeled monoclonal antibody (Figure 1) with several fluorophores.

**Figure 3:**
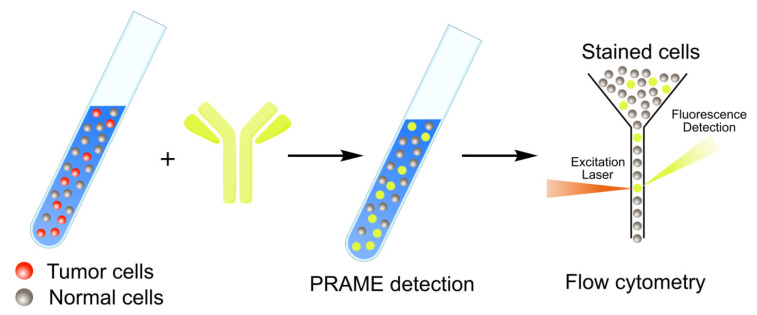
Flow cytometry approach to PRAME detection using fluorescent antibodies.

The obtained fluorescent antibodies were applied to a quantitative analysis of PRAME expression in the model cell lines (K562 and AMO-1) using a flow cytometry approach (Figure 3).

**Figure 4:**
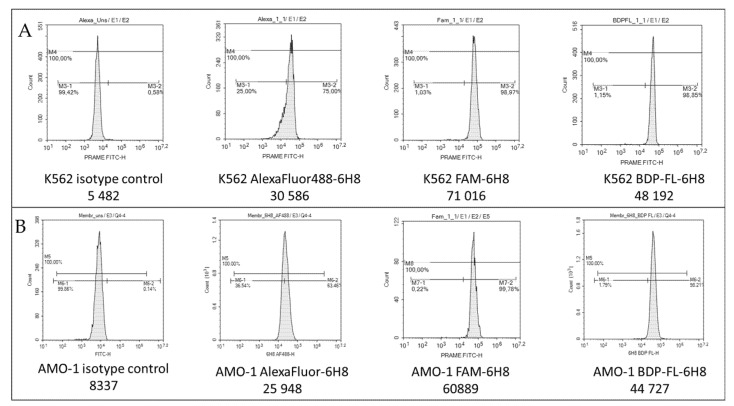
Flow cytometry of model cell lines K562 (**A**) and AMO-1 (**B**).

We obtained data for the staining of K562 and AMO-1 cells with PRAME-recognizing antibodies. As a result of staining with antibodies labeled with fluorescent molecules, the cells of these lines acquired significantly higher fluorescence parameters compared to cells stained with an isotypic control (Figure 4).

The *PRAME* expression level in the K562 line was found to be 1393 ± 83% relative to *ABL* (Abelson kinase gene). The *PRAME* expression level in the AMO-1 line was 414 ± 29% relative to ABL. Thus, the presence of the PRAME protein on the surface of K562 and AMO-1 cells was confirmed. It should be noted that the K562 line is widely used as a model for cells overexpressing PRAME. There exist a number of reports of high *PRAME* mRNA levels in these cells [45,46,47,48,49,50,51,52,53,54]. Moreover, the results of staining the K562 cell membrane with PRAME-recognizing antibodies have been previously investigated [32,55]. Our results are consistent with previous observations, indicating the occurrence of binding of PRAME-recognizing antibodies to the surface of a PRAME-expressing cell.

To the best of our knowledge, studies on *PRAME* activity in AMO-1 cells, a model of multiple myeloma, have not yet been conducted. We chose this line for research due to the fact that multiple myeloma is characterized by a high frequency of *PRAME* expression [56]. The activity of the *PRAME* gene and the intensity of staining of the AMO-1 membrane were lower compared to K562 cells (Figure 4). In general, this is not surprising, since the amount of mRNA of the *PRAME* gene, and of the content of the protein that it encodes within the cell is of a direct ratio [57]. Therefore, a lower *PRAME* RNA level in the cell results in a lower content of the mature protein.

The highest intensity of cell staining was observed when using antibodies labeled with BDP-FL and FAM dyes. A slightly lower intensity was observed when using the AF488 dye. Encouraged by these results, we used antibodies labeled with the BDP-FL dye to study the bone marrow of patients with oncohematological diseases.

Blast cells from patients with acute myeloid leukemia (AML) were also successfully stained with antibodies to PRAME (Table 1).

There is a tendency towards a more intense staining of blast cells in patients with a higher level of *PRAME* expression. At the same time, the fluorescence of lymphocytes in the studied samples did not change. Furthermore, an increase in the fluorescence of blast cells did not occur in AML patients in remission, with blast cells of a normal immunophenotype.

The results obtained indicate the success of the modification of the 6H8 antibody with fluorescent labels. Antibodies can be used in the routine diagnostic practice of laboratories that perform immunophenotyping of bone marrow in patients with acute leukemia. Data on *PRAME* expression on the surface of leukemic cells can be useful for planning PRAME-specific therapy.

### 2.4. Confocal Microscopy of K562 Cells Stained with AF488-Labeled 6H8 Monoclonal Antibody 

**Figure 5:**
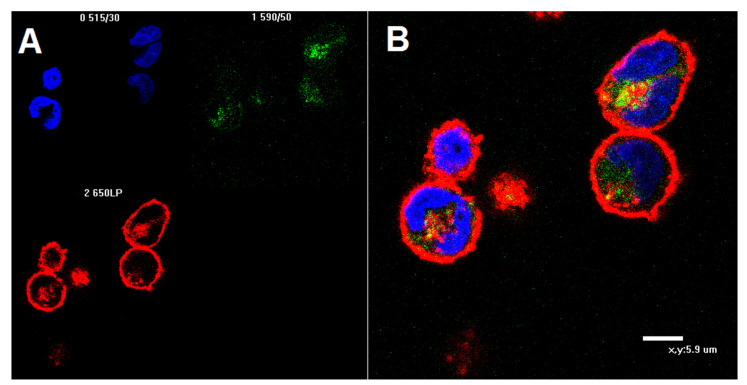
Confocal microscopy of K562 cells stained with AF488-labeled 6H8 antibody (green), DAPI (blue), and PKH26 (red). Single channel images (**A**), and merged channels (**B**).

To visualize the binding of the labeled 6H8 antibody with the PRAME antigen, a confocal microscopy of stained cells was performed (Figure 5). The standard K562 cell line, which contains a surface form of PRAME protein, was selected for observation. Labeling was performed on live K562 cells prior to fixation using paraformaldehyde, to prevent permeabilization and the binding of the AF488-labeled antibody 6H8 with the intracellular form of the PRAME protein. The membrane structures and nucleus were stained with PKH26 and DAPI tracers, correspondingly.

The incubation of K562 cells with the AF488-labeled 6H8 antibody led to staining of the PRAME protein on the surface of cells and in the cytoplasm. These results suggest that the dye-modified antibody internalizes into the cell as a result of the endocytosis mechanism.

## 3. Materials and Methods

### 3.1. General Methods

Dimethylformamide (DMF) was dried over CaH_2_, then distilled under reduced pressure over P_2_O_5_ and kept under argon in the dark. Tetrahydrofuran (THF), dioxane and diethyl ether were distilled over sodium-benzophenone under argon and kept in the dark under an inert atmosphere. Ethyl N-(11-azido-3,6,9-trioxaundecyloxy) acetimidate **1** was prepared as described [42]; NHS derivatives of 6-carboxyfluorescein, 6-carboxy-Alexa488, and BDP-FL were obtained from Lumiprobe. The 6H8 monoclonal antibody was obtained as described in a previous study [58]. All NMR data for the synthesized compounds were obtained in DMSO-*d*_6_ solution at 30 °C. The 1D ^1^H, ^13^C, and ^15^N NMR spectra were acquired using a Bruker AVANCE-III-600 spectrometer equipped with a room-temperature probe. MS spectra were acquired using Thermo Orbitrap Elite hybrid instrument using a Thermo Accela UPLC system, equipped with a Phenomenex Aeris XB-C8 widepore column (150 × 2.1 mm, 3.6 μm). Samples were eluted with a 5→85% gradient of MeCN in water, with 0.1% formic and 0.02% trifluoroacetic acids as the eluent additive. Detection was achieved by UV-VIS DAD and full scan MS (ESI+/−, 250–2000 au).

### 3.2. Synthetic Procedures

**Ethyl N-(11-amino-3,6,9-trioxaundecyloxy)acetimidate** (**2**). Triphenylphosphine (550 mg; 2.1 mmol) was added in one portion to a vigorously stirred solution of ethyl N-(11-azido-3,6,9-trioxaundecyloxy)acetimidate (**1**) (532 mg; 1.75 mmol) in dry THF (15 mL), and stirring was continued for 2 h. Then, water (500 µL) was added, and the reaction mixture was refluxed until the intermediate, phosphazene, was consumed (TLC control in 5% methanol in DCM, phosphazene *R*_f_ 0.7). After the evaporation of the solvent in vacuum, the residue was chromatographed on silica gel (Et_3_N/MeOH/DCM, gradient of Et_3_N from 0 to 5%). The desired compound **2** was obtained as a yellowish oil, with a yield of 423 mg (87%). *R*_f_ 0.42 (5% MeOH/5% Et_3_N/DCM). ^1^H NMR (DMSO-*d*_6_): 3.98–3.91 (m, 4H), 3.61–3.47 (m, 12H), 3.36 (t, *J* = 5.56 Hz, 2H), 2.65 (t, *J* = 5.79 Hz, 2H), 1.87 (s, 3H) and 1.21 (t, *J* = 7.05 Hz, 3H). ^13^C NMR (DMSO-*d_6_*): 178.88, 162.18, 73.28, 72.84, 70.37, 70.31, 70.28, 70.08, 68.98, 62.28, 14.69 and 13.93. HRMS: 279.1923 (calc. for C_12_H_26_N_2_O_5_ [M+H]^+^ 279.1920).

**General procedure for ethoxyethylidene protected oxyamine derivatives 3**,**4**,**5**. Amine **2** (22.2 mg; 0.08 mmol) was added to a solution of dye NHS-ester (0.07 mmol) in dry DMF (15 mL) at 0 °C and vigorous stirring was performed. Then, DIPEA (36.5 µL, 0.21 mmol) was added dropwise to the reaction. The reaction was monitored by TLC. After the consumption of the starting NHS ester, the reaction mixture was evaporated to achieve dryness *in vacuo*, and the residue was chromatographed on an appropriate sorbent using an appropriate solvent system. The following compounds were synthesized:

**1,3-Dimethyl-5-{2-[11-(1-ethoxyethylideneaminooxy)-3,6,9-trioxaundecylamino¬carbonyl]ethyl}-4,4-difluoro-4-bora-3a,4a-diaza-s-indacene** (**3**) was purified by column chromatography on silica gel using gradient elution with 0→5% EtOH in DCM. The desired compound was obtained as a dark orange oil, at a yield of 25.9 mg (67%). *R*_f_ 0.45 (5% EtOH in DCM). ^1^H NMR (DMSO-*d*_6_): 7.95 (s, 1H), 7.09 (s, 1H), 6.36 (s. 1H), 6.29 (s, 1H), 3.96–3.92 (m, 2H), 3.63–3.47 (m, 12H), 3.25–3.21 (m, 2H), 3.12–3.06 (m, 2H), 2.48–2.44 (m, 5H), 2.25 (s, 3H), 1.86–1.83 (m, 2H), 1.8–1.73 (m, 2H) and 1.22–1.17 (m, 2H). ^13^C NMR (DMSO-*d*_6_): 179.88, 171.43, 162.20, 159.57, 158.37 144.49, 133.52, 132.84, 131.98, 129.17, 125.71, 120.70, 117.08, 72.83, 72.45, 70.23, 69.57, 68.97, 62.27, 51.69, 34.19, 29.52, 24.48, 14.92, 13.90 and 11.41. HRMS: 553.3015 (calc. for C_26_H_39_BF_2_N_4_O_6_ [M+H]^+^ 553.3003).

**6-[11-(1-Ethoxyethylideneaminooxy)-3,6,9-trioxaundecylaminocarbonyl]}fluores¬cein (4)** was purified by column chromatography on silica gel using gradient elution with 0→5% EtOH in DCM. The desired compound was obtained as a yellow oil. The yield was 26.7 mg (60%). *R*_f_ 0.5 (5% EtOH in DCM). ^1^H NMR (DMSO-*d*_6_): 10.13 (br s, 1H), 8.69 (t, *J* = 5.43 Hz, 1H), 8.19–8.14 (m, 1H), 8.07 (d, *J* = 7.92 Hz, 1H), 7.69 (s, 1H), 6.72–6.68 (m, 2H), 6.61–6.54 (m, 4H), 3.93 (q, *J* = 7.04 Hz, 2H), 3.92–3.88 (m, 2H), 3.57–3.53 (m, 2H), 3.51–3.44 (m, 12H), 3.38–3.35 (m, 2H), 1.85 (s, 3H) and 1.20 (t, *J* = 7.04 Hz, 3H). ^13^C NMR (DMSO-*d*_6_): 173.15, 168.50, 165.12, 162.18, 160.10, 153.15, 152.35, 141.12, 129.87, 129.64, 128.69, 125.29, 122.77, 113.22, 109.70, 102.76, 83.85, 72.79, 70.30, 70.25, 70.21, 70.16, 70.02, 69.10, 68.93, 62.26. 25.70, 15.64, 14.79, 14.66, 14.37 and 13.90. HRMS: 637.2397 (calc. for C_33_H_36_N_2_O_11_ [M+H]^+^ 637.2392).

**3,6-Diamino-4,5-disulfo-9-{2-carboxy-5-[11-(1-ethoxyethylideneaminooxy)-3,6,9-trioxaundecylaminocarbonyl]phenyl}xanthylium (5)** was purified by column chromatography in silica gel gradient elution with 0→25% EtOH in DCM. The desired compound was obtained as a dark red powder. The yield was 44.5 mg (80%). Due to low stability of Alexa 488, the compound was used for antibody labeling without further purification. *R*_f_ 0.36 (56:40:3:1, DCM/MeOH/H_2_O/Et_3_N). ESI MS: 793.2 (calc. for C_33_H_37_N_4_O_15_S_2_ [M]^−^ 793.2).

### 3.3. Antibody Staining

The modification of the 6H8 antibody was initiated by periodate oxidation in an acidic acetate buffer (pH 5) [23]. The reaction was carried out for 1 h at room temperature in the dark. The excess of sodium periodate was quenched with a glycerol solution and the oxidized antibody was desalted using a Sephadex G-50 column and an acidic acetate buffer with pH 3.6. The subsequent reaction with protected oxyamines **3**–**5** for 1 h produced fluorescently labeled antibodies. The reagents **3**–**5** have an unlimited storage time. The ethoxyethylydene protecting group was removed in situ in an acidic medium and instantly reacted with carbonyl groups of the oxidized antibody. The excess dye was removed using gel chromatography on Sephadex G-50 in a PBS buffer.

### 3.4. Cell Line Handling

Cultures of cell lines K562 (chronic myeloid leukemia) and AMO-1 (multiple myeloma) were incubated at +37 °C, in a CO_2_ incubator on an RPMI-1640 medium containing 2 mM glutamine, 10 mM HEPES, 10% fetal bovine serum (FBS) and 40 μg/mL gentamicin sulfate. The cells were subcultured twice weekly in a new medium at a concentration of 100,000 cells per mL.

### 3.5. Bone Marrow Sample Collection

Bone marrow samples from patients with acute myeloid leukemia were provided by GeneTechnology, (Moscow, Russia). Each patient signed an informed medical consent form confirming their voluntary participation in the scientific research.

### 3.6. RNA Isolation and Real-Time PCR

The isolation of total RNA from cell lines and bone marrow materials of patients with acute myeloid leukemia was performed using the “RNA-extract” reagent set (GeneTechnology, Moscow, Russia), in accordance with the guidelines provided by the vendor. Subsequent synthesis of cDNA and the determination of expression levels of the PRAME gene and the Abelson kinase (ABL) housekeeping gene were performed using Oncoscreen-9Q and Oncoscreen-14Q kits (GeneTechnology, Moscow, Russia), respectively, according to the guidelines provided by the vendor. Real-time PCR was performed on a DT-Lite device (DNA-Technology, Moscow, Russia). The level of PRAME expression was assessed using a program provided by DNA-technology (Moscow, Russia).

### 3.7. Cells Staining and Flow Cytometry

K562 or AMO-1 cells (1 million), or 500 μL of patients’ bone marrow, were used for flow cytometry. Both the K562 and AMO-1 cells were washed from the culture medium by centrifugation in a PBS solution for 5 min at 500 rpm. Bone marrow samples were subjected to hemolysis in 5 mL of 0.9% NH_4_Cl solution for 10 min to remove erythrocytes. Then, the cells were twice washed by centrifugation in PBS solution for 5 min at 500 rpm. The K562 and AMO-1 cells, as well as the patients’ bone marrow cells, were resuspended in 1 mL of PBS, and 100 μL samples were used for staining with antibodies to PRAME and the isotypic control. The samples were treated with 0.1–0.5 μL of a 1 mg/mL solution of labeled antibodies. Additionally, bone marrow cells were stained with antibodies to antigens CD45, CD34, CD117, CD13, CD33, CD14, CD36, CD15, CD11c, CD41a, MPO, CD19, CD22, CD10, CD2, CD3, CD4, CD8, CD56 and CD15 (SonyBiotechnology, San Jose, CA, USA) to determine blast cell counts, as well as the number of normal lymphocytes and granulocytes. Incubation with antibodies was conducted for 15 min in the dark. After incubation, unbound antibodies were washed off and cells were analyzed on an ACEA NovoCyte flow cytometer (ACEA Biosciences, San Diego, CA, USA).

Cells of tumor lines and samples obtained from patients were stained with antibodies to the PRAME antigen, and the fluorescence of the cells was measured in comparison to the control stained with isotypic antibodies. In bone marrow samples from patients with acute leukemia, the degree of staining of blast cells and other cells with a normal immunophenotype was also assessed.

### 3.8. Cell Staining for Confocal Microscopy

K562 cells were transferred into a PBS-BSA buffer and incubated with AF488-labeled monoclonal antibody 6H8 at 37 °C. After 1 h, the cells were precipitated by centrifugation, resuspended in PBS, and incubated with 3.7% paraformaldehyde in PBS for 15 min at room temperature. The fixed cells were washed with PBS and stained for 20 min with DAPI, and for 10 min with a PKH26 tracker. Then, the staining medium was removed, and the cells were washed with PBS again. The coverslips were placed onto the slides with a drop of 10% Mowiol in 0.1 M Tris-HCl pH 8.5 and washed stained K562 cells. Slides were analyzed using an Eclipse TE2000 confocal microscope (Nikon, Tokyo, Japan) with 515/30 (blue), 590/50 (green), and red (650LP) filters sets.

## 4. Conclusions

This study aimed to contribute to the development of a facile method for fluorescent modification of antibodies with dyes containing a masked oxyamine group. After synthesis, these modification reagents can be stored for an unlimited time, thus allowing the rapid and convenient labeling of all types of immunoglobulin G. Our approach utilizes periodate oxidation as the first step, and can therefore potentially be applied to any glycosylated antibody.

The synthetic procedure was applied to a PRAME-specific monoclonal 6H8 antibodies, yielding a fluorescent immunoconjugate that was found to be effective for PRAME detection, both in model cell lines and in bone marrow samples of patients with acute leukemia. The constructed fluorescent probe allowed us to investigate *PRAME* expression in the AMO-1 cell line. Moreover, the PRAME-specific probe provided reliable data on bone marrow samples, thus, it can be considered as a potential and promising tool for prognostic and diagnostic purposes.

## Figures and Tables

**Table 1:** Staining the bone marrow cells populations from AML patients; data are given in median fluorescence intensity (MFI) units.

Patient	*PRAME* Gene Expression Level (% Relative to *ABL*)	Blast Cell Fluorescence		Lymphocyte Fluorescence	
		Isotypic Control	BDP-FL-6H8	Isotypic Control	BDP-FL-6H8
M2	255	324	6872	113	124
M4-53	13	352	580	1453	1428
M4-40	378	295	8656	1135	1156

## Data Availability

Not applicable.
